# Supplementary material for: Biomass fuel use and birth weight among term births in Nigeria
Source: PLOS Glob Public Health. 2022 Jun 10;2(6):e0000419. doi: 10.1371/journal.pgph.0000419 (PMC10022098; doi:10.1371/journal.pgph.0000419)
Supplement: S1 Table — (DOCX) [file pgph.0000419.s001.docx]

**S1 Table. Sensitivity analysis: 2018 Nigeria DHS maternal and infant characteristics stratified by participation status***

| **Characteristics** | **Overall** | **Participant*** | **Non-Participant** | **p-value** |
| --- | --- | --- | --- | --- |
| n | 33924 | 6975 (20.6) | 26949 (79.4) |  |
| **Type of cooking fuel** |  |  |  |  |
| Low pollution fuel | 2487 | 1615 (64.9) | 872 (35.1) | <0.01 |
| Kerosene | 2674 | 1411 (52.8) | 1263 (47.2) |  |
| Biomass fuel | 28084 | 3949 (14.1) | 24135 (85.9) |  |
| **Region** |  |  |  |  |
| North-central | 5875 | 1333 (75.6) | 4442 (75.6) | <0.01 |
| North-west | 10305 | 571 (5.5) | 9734 (94.5) |  |
| North-east | 7211 | 509 (7.1) | 6702 (92.9) |  |
| South-east | 3798 | 1758 (46.3) | 2040 (53.7) |  |
| South-south | 3202 | 1074 (33.5) | 2128 (66.5) |  |
| South-west | 3533 | 1630 (46.1) | 1903 (53.9) |  |
| **Type of place of residence** |  |  |  |  |
| Urban | 11699 (34.5) | 4246 (60.9) | 7453 (27.7) | <0.01 |
| Rural | 22225 (65.5) | 2729 (39.1) | 19496 (72.3) |  |
| **Number of household members, mean (SD)** |  |  |  |  |
| <5 | 8826 (26.0) | 2522 (36.2) | 6304 (23.4) | <0.01 |
| 5-9 | 9939 (29.3) | 2508 (36.0) | 7431 (27,6) |  |
| ≥10 | 15159 (44.7) | 1945 (27.9) | 13214 (49.0) |  |
| **Maternal age** |  |  |  |  |
| <20 | 15391 (45.4) | 435 (6.2) | 3734 (13.9) | <0.01 |
| 20-34 | 5274 (15.5) | 5397 (77.4) | 18892 (70.1) |  |
| ≥35 | 5466 (16.1) | 1143 (16.4) | 4323 (16.0) |  |
| **Maternal education** |  |  |  |  |
| None | 15391 (45.4) | 577 (8.3) | 14814 (55.0) | <0.01 |
| Primary | 5274 (15.5) | 906 (13.0) | 4368 (16.2) |  |
| Secondary | 10623 (31.3) | 3778 (54.2) | 6845 (25.4) |  |
| Higher | 2636 (7.8) | 1714 (24.6) | 922 (3.4) |  |
| **Birth order (parity)** |  |  |  |  |
| 1 | 6476 (19.1) | 1798 (25.8) | 4678 (17.4) | <0.01 |
| 2 | 6123 (18.0) | 1573 (22.6) | 4550 (16.9) |  |
| 3 | 5274 (15.5) | 1302 (18.7) | 3972 (14.7) |  |
| 4+ | 16051 (47.3) | 2302 (33.0) | 13749 (51.0) |  |
| **Place of delivery** |  |  |  |  |
| Health facility | 13975 (41.2) | 6418 (92.0) | 7557 (28.0) | <0.01 |
| Home | 19949 (58.8) | 557 (8.0) | 19392 (72.0) |  |
| **Type of delivery** |  |  |  |  |
| Caesarean section | 879 (2.6) | 493 (7.1) | 386 (1.4) | <0.01 |
| Vaginal | 32899 (97.4) | 6409 (92.9) | 26490 (98.6) |  |
| **Child sex** |  |  |  |  |
| Male | 17257 (50.9) | 3569 (51.2) | 13688 (50.8) | 0.58 |
| Female | 16667 (49.1) | 3406 (48.8) | 13261 (49.2) |  |
| **Size at birth** |  |  |  |  |
| Below average | 4572 (13.7) | 601 (8.6) | 3971 (15.0) | <0.01 |
| Average+ | 28826 (86.3) | 6364 (91.4) | 22462 (85.0) |  |
| **Source of information about birthweight** |  |  |  |  |
| Not weighed | 23992 (70.7) | 0 | 23992 (89.0) |  |
| From written card | 2906 (8.6) | 2668 (38.3) | 238 (0.9) |  |
| From mother’s recall | 4822 (14.2) | 4307 (61.7) | 515 (1.9) |  |
| Don’t know | 2204 (6.5) | 0 | 2204 (8.2) |  |

*Participant = Women that gave birth in the last 5 years with data on birthweight, cooking fuel type and other selection criteria.

DHS=Demographic and Health Survey

P-value = Difference in proportions within categories of maternal and infant characteristics between participants and non-participants.
