# Supplementary material for: Biomass fuel use and birth weight among term births in Nigeria
Source: PLOS Glob Public Health. 2022 Jun 10;2(6):e0000419. doi: 10.1371/journal.pgph.0000419 (PMC10022098; doi:10.1371/journal.pgph.0000419)
Supplement: S2 Table — (DOCX) [file pgph.0000419.s002.docx]

**S2 Table. Sensitivity analysis: 2018 Nigeria DHS maternal and infant characteristics stratified by sources of information about birth weight (written card Vs mother’s recall)***

| Characteristics | Overall | Written Card | Mother’s recall | p-value** |
| --- | --- | --- | --- | --- |
| n | 6975 | 2668 (38.3) | 4307 (61.7) |  |
| Type of place of residence |  |  |  |  |
| Urban | 4246 (60.9) | 1528 (57.3) | 2718 (63.1) | <0.01 |
| Rural | 2729 (39.1) | 1140 (42.7) | 1589 (36.9) |  |
| Number of household members |  |  |  |  |
| <5 | 2522 (36.2) | 956 (35.8) | 1566 (36.4) | 0.04 |
| 5-9 | 2508 (36.0) | 924 (34.6) | 1584 (36.8) |  |
| ≥10 | 1945 (27.9) | 788 (29.5) | 1157 (26.9) |  |
| Wealth index (item count) |  |  |  |  |
| 0-6 | 850 (12.2) | 355 (13.3) | 495 (0.0) | 0.07 |
| 7-12 | 2523 (36.2) | 963 (36.1) | 1560 (36.2) |  |
| 13-24 | 3602 (51.6) | 1350 (50.6) | 2252 (52.3) |  |
| Maternal age, years |  |  |  |  |
| <20 | 435 (6.2) | 181 (6.8) | 254 (5.9) | 0.24 |
| 20-34 | 5397 (77.4) | 2064 (77.4) | 3333 (77.4) |  |
| ≥35 | 1143 (16.4) | 423 (15.9) | 720 (16.7) |  |
| Maternal education |  |  |  |  |
| None | 577 (8.3) | 265 (9.9) | 312 (7.2) | <0.01 |
| Primary | 906 (13.0) | 398 (14.9) | 508 (11.8) |  |
| Secondary | 3778 (54.2) | 1440 (54.0) | 2338 (54.3) |  |
| Higher | 1714 (24.6) | 565 (21.2) | 1149 (26.7) |  |
| Birth order (parity) |  |  |  |  |
| 1 | 1798 (25.8) | 684 (25.6) | 1114 (25.9) | 0.73 |
| 2 | 1573 (22.6) | 610 (22.9) | 963 (22.4) |  |
| 3 | 1302 (18.7) | 482 (18.1) | 820 (19.0) |  |
| 4+ | 2302 (33.0) | 892 (33.4) | 1410 (32.7) |  |
| Place of delivery |  |  |  |  |
| Health facility | 6418 (92.0) | 2414 (90.5) | 4004 (93.0) | <0.01 |
| Home | 557 (8.0) | 254 (9.5) | 303 (7.0) |  |
| Type of delivery |  |  |  |  |
| Caesarean section | 493 (7.1) | 178 (6.8) | 315 (7.4) | 0.32 |
| Vaginal | 6409 (92.9) | 2459 (93.2) | 3950 (92.6) |  |
| Child sex |  |  |  |  |
| Male | 3569 (51.2) | 1386 (51.9) | 2183 (50.7) | 0.31 |
| Female | 3406 (48.8) | 1282 (48.1) | 2124 (49.3) |  |
| Size at birth |  |  |  |  |
| Below average | 601 (8.6) | 219 (8.2) | 382 (8.9) | 0.34 |
| Average+ | 6364 (91.4) | 2444 (91.8) | 3920 (91.1) |  |
| Birthweight (g), mean (SD) | 3291 642) | 3284 (623) | 3295 (653) | 0.49 |

*Dataset of children of women that gave birth in the last 5 years with data on birthweight and cooking fuel type. DHS=Demographic and Health Survey

**P-value for difference in proportions within categories of maternal and infant characteristics by sources of information about birthweight from written card and mother’s recall.
