# Supplementary material for: Biomass fuel use and birth weight among term births in Nigeria
Source: PLOS Glob Public Health. 2022 Jun 10;2(6):e0000419. doi: 10.1371/journal.pgph.0000419 (PMC10022098; doi:10.1371/journal.pgph.0000419)
Supplement: S3 Table — (DOCX) [file pgph.0000419.s003.docx]

**S3 Table. Association between covariates and birth weight (in grams) among term births, results of linear regression from CEGROMS (N=1514)**

| **Predictor** | **N** | **Least square mean birth weight (SE)** | **β (95% confidence Interval)** |
| --- | --- | --- | --- |
| **Maternal age** |  |  |  |
| <20 years | 48 | 3165 (75) | Reference |
| 20-34 years | 1151 | 3168 (32) | 2.5 (-137.1, 142.1) |
| ≥35 years | 315 | 3147 (41) | -18.5 (-172, 135) |
| **Birth order (parity)** |  |  |  |
| 1 | 429 | 3071 (42) | Reference |
| 2 | 376 | 3157 (44) | 85.5 (20.4, 150.6) |
| 3 | 275 | 3229 (46) | 157.8 (85.5, 230) |
| 4+ | 434 | 3183 (40) | 112.4 (40.9, 184) |
| **Maternal education level** |  |  |  |
| Tertiary | 782 | 3143 (35) | Reference |
| Secondary | 635 | 3130 (31) | -13.3 (-66.4, 39.8) |
| Primary | 74 | 3096 (60) | -47.4 (-169.1, 74.3) |
| None | 23 | 3271 (101) | 128.2 (-75.9, 332.2) |
| **Body mass index** |  |  |  |
| Normal weight (<25 kg/m^2^) | 644 | 3069 (39) | Reference |
| Overweight (25.0-29.9 kg/m^2^) | 496 | 3172 (41) | 103.1 (48.1, 158) |
| Obese (≥30 kg/m^2^) | 374 | 3238 (44) | 168.7 (107.8, 229.7) |
| **Child sex** |  |  |  |
| Male | 783 | 3229 (39) | Reference |
| Female | 731 | 3091 (39) | -137.3 (-184, -90.6) |

*β= Difference in birth weight (in grams) for exposure to kerosene and biomass fuel relative to LPG by category of maternal age, birth order (parity) and maternal educational level. Adjusted for maternal age, birth order (parity), educational level, BMI, and child sex. CI = Confidence interval.
