# Supplementary material for: Biomass fuel use and birth weight among term births in Nigeria
Source: PLOS Glob Public Health. 2022 Jun 10;2(6):e0000419. doi: 10.1371/journal.pgph.0000419 (PMC10022098; doi:10.1371/journal.pgph.0000419)
Supplement: S4 Table — (DOCX) [file pgph.0000419.s004.docx]

**S4 Table. Associations between biomass fuel and birth weight by levels of other factors in 2018 Nigeria Demographic and Health Survey (N=6975)**

| **Predictor** | **N** | **Least square mean birth weight, g (SE)** | **β (95% confidence Interval)** |
| --- | --- | --- | --- |
| **Community level factors** |  |  |  |
| **Region** |  |  |  |
| North-central | 1433 | 3270 (28) | Reference |
| North-west | 571 | 3025 (37) | -245.5 (-318.6, -172.4) |
| North-east | 509 | 3291 (39) | 20.7 (-55.5, 96.9) |
| South-east | 1758 | 3417 (30) | 146.9 (90, 203.9) |
| South-south | 1074 | 3378 (30) | 107.6 (46.2, 169) |
| South-west | 1630 | 3234 (27) | -35.9 (-93, 21.3) |
| **Type of place of residence** |  |  |  |
| Urban | 2649 | 3244 (23) | Reference |
| Rural | 2264 | 3294 (25) | 50.1 (9.5, 90.7) |
| **Household level factors** |  |  |  |
| **Number of household members** |  |  |  |
| <5 | 2522 | 3257 (24) | Reference |
| 5-9 | 2508 | 3270 (25) | 13.1 (-27.9, 54) |
| ≥10 | 2061 | 3280 (27) | 23.5 (-23.9, 70.8) |
| **Wealth index (item count)** |  |  |  |
| ≥6 | 838 |  | Reference |
| 7-12 | 2523 | 3223 (31) | 53.2 (-1.2, 107.5) |
| 13-24 | 3602 | 3278 (25) | 83.1 (24.5, 141.7) |
| **Individual level factors** |  | 3307 (23) |  |
| **Maternal age, years** |  |  |  |
| <20 | 435 | 3222 (37) | Reference |
| 20-34 | 5397 | 3305 (20) | 83.7 (17.9, 149.4) |
| >35 | 1143 | 3280 (27) | 58.3 (-20.8, 137.4) |
| **Maternal education** |  |  |  |
| Tertiary | 1714 | 3344 (26) | Reference |
| Secondary | 3778 | 3319 (23) | -24.8 (-67.2, 17.5) |
| Primary | 906 | 3243 (30) | -101.4 (-162, -40.7) |
| No education | 577 | 3171 (34) | -173.5 (-245.3, -101.6) |
| **Birth order (parity)** |  |  |  |
| 1 | 4673 | 3211 (25) | Reference |
| 2 | 2302 | 3260 (26) | 48.9 (10, 87.8) |
| 3 | 2302 | 3272 (27) | 60.5 (13.7, 107.4) |
| 4+ | 2302 | 3334 (25) | 122.2 (72.8, 171.7) |
| **Child sex** |  |  |  |
| Male | 3569 | 3324 (23) | Reference |
| Female | 3406 | 3215 (23) | -109.2 (-137.2, -81.2) |
| **Delivery method** |  |  |  |
| Vaginal | 3569 | 3185 (18) | Reference |
| C-section | 3406 | 3354 (33) | 169.2 (109.6, 228.7) |

BW=Birth weight. *β= Difference in BW (in grams) for exposure to a kerosene and biomass fuel relative to low pollution fuel. Adjusted for the hierarchy of covariates.
