# Supplementary material for: Biomass fuel use and birth weight among term births in Nigeria
Source: PLOS Glob Public Health. 2022 Jun 10;2(6):e0000419. doi: 10.1371/journal.pgph.0000419 (PMC10022098; doi:10.1371/journal.pgph.0000419)
Supplement: S5 Table — (DOCX) [file pgph.0000419.s005.docx]

**S5 Table. Association between biomass fuel use and birth weight by sex within regions in Nigeria: DHS 2018**

| **Variable** | **β (95% confidence Interval)** | **Interaction P-value‡** |
| --- | --- | --- |
| **Northcentral** Male | -66 (-185 to 52) | 0.09 |
| Female | 50 (-73 to 173) |  |
| **Northwest** Male | -113 (-294 to 68) | 0.57 |
| Female | -57 (-248 to 134) |  |
| **Northeast** Male | -322 (-591 to -52) | 0.38 |
| Female | -141 (-476 to 193) |  |
| **Southeast** Male | -17 (-115 to 81) | 0.97 |
| Female | -19 (-117 to 79) |  |
| **South-South** Male | -93 (-231 to 46) | 0.48 |
| Female | -148 (-287 to -9) |  |
| **Southwest** Male | -29 (-133 to 74) | 0.03 |
| Female | 99 (-9 to 207) |  |

**‡Interaction P-value:** The interaction effects were tested using likelihood-ratio test. The variables were treated as categorical and the degrees of freedom of each test degrees of freedom of each test are number of levels of each variable minus one.
