# Supplementary material for: Biomass fuel use and birth weight among term births in Nigeria
Source: PLOS Glob Public Health. 2022 Jun 10;2(6):e0000419. doi: 10.1371/journal.pgph.0000419 (PMC10022098; doi:10.1371/journal.pgph.0000419)
Supplement: Attachment — Submitted filename: Authors response to reviews_Feb 17 2022 SJL.doc [file pgph.0000419.s010.doc]

**Author's response to reviews**

**Title: Biomass fuel use and birth weight among term births in Nigeria**

**Authors:**

1. Musa Abubakar Kana – [musakana77@yahoo.com](mailto:musakana77@yahoo.com)
2. Min Shi – [shi2@niehs.nih.gov](mailto:shi2@niehs.nih.gov)
3. Jennifer Ahmed – [kyenbo@gmail.com](mailto:kyenbo@gmail.com)
4. Jimoh Muhammad Ibrahim – [drjim20032000@yahoo.com](mailto:drjim20032000@yahoo.com)
5. Abdullahi Yusuf Ashir – [abdull.yusuf24@gmail.com](mailto:abdull.yusuf24@gmail.com)
6. Karima Lami Abdullahi – [karimatu2004@gmail.com](mailto:karimatu2004@gmail.com)
7. Halima Bello-Manga - [mamanzara2@gmail.com](mailto:mamanzara2@gmail.com)
8. Matthew Taingson – [taingson@yahoo.com](mailto:taingson@yahoo.com)
9. Amina Mohammed-Durosinlorun - [aminamhmd4@gmail.com](mailto:aminamhmd4@gmail.com)
10. Musa Shuaibu – [aminamhmd4@gmail.com](mailto:aminamhmd4@gmail.com)
11. Abdullkadir Musa Tabari - [musatabari@gmail.com](mailto:musatabari@gmail.com)
12. Stephanie J. London - [london2@niehs.nih.gov](mailto:london2@niehs.nih.gov)

17^th^ February 2022

The Editor-in-Chief

PLOS Global Public Health

PGPH-D-21-00999: **Intimate partner violence during pregnancy is related to reduced birthweight: cross-sectional study in Kaduna, Northwestern Nigeria.**

Musa Abubakar Kana, MBBS, PhD; Min Shi, PhD; Jennifer Ahmed, HND; Abdullahi Yusuf Ashir, BSc; Karima Lami Abdullahi, PhD; Halima Bello-Manga, MBBS, MPH, FMCPath; Matthew Taingson, MBBS, FMCOG; Amina Mohammed-Durosinlorun, MBBS, FWACS, FMCOG; Musa Shuaibu, MBBS, FMCPaed; Abdullkadir Musa Tabari, MBBS, FMCR; Stephanie London, MD, DrPH

--------------------------------------------------------------------------------

**Editorial and Journal requirements**

1. Please amend your detailed Financial Disclosure statement. This is published with the article, therefore should be completed in full sentences and contain the exact wording you wish to be published.

**Response:** We have amended the Financial Disclosure statement in the manuscript completed in full sentences and contain the exact wording we wish to be published.

2. Please state what role the funders took in the study. If the funders had no role in your study, please state: “The funders had no role in study design, data collection and analysis, decision to publish, or preparation of the manuscript.”

**Response:** We have edited the manuscript and indicated the role the funders took in the study.

3. Please ensure that the funders and grant numbers match between the Financial Disclosure field and the Funding Information tab in your submission form. Note that the funders must be provided in the same order in both places as well.

**Response:** We have ensured that the funders and grant numbers matched both the Financial Disclosure field and the Funding Information tab in our submission form.

4. Please update your Competing Interests statement. If you have no competing interests to declare, please state: “The authors have declared that no competing interests exist.”

**Response:** We have edited the manuscript and updated the Competing Interests statement accordingly.

5. Please provide separate figure files in .tif or .eps format only and ensure that all files are under our size limit of 20MB.

**Response:** We have provided separate figure files in .tif or .eps formats and ensured that all the files are under size limit of 20MB.

**Referee comments to Author**

1. Please check your objective and check why it is different from other studies that used similar analytic approaches to examine the relations of maternal exposure to biomass fuel and birth weight that have already identified in a study conducted by Jiang et al (2016) published in BMC public health.

**Response:** We believe that the reviewer is referring to Jiang et al. 2015 which is ref #16 in our manuscript. We referred to this paper twice. However, the first citation might not have been very clear. So, we have revised that sentence in the introduction to clarify this point: “However, it is unclear if this reduced birth weight or size is due to preterm birth or intrauterine growth restriction, as few studies, including one from China^16^, but none from sub-Saharan Africa (SSA) adjusted for gestational age.” In the discussion we refer again to Jiang et al. but put the numeric reference after the second sentence making the reference less clear. So we have revised the sentence in the discussion to say that “In a Chinese birth cohort study that examined the association of biomass fuel and birth weight as a binary variable (low weight versus normal) a significant negative association was seen only in preterm births ^16^. However, power was limited for analysis of biomass among term births with only 7 exposed low birth weight babies^16^“.

2. Justify why data of 2018 was used instead of multiple years.

**Response:** The 2018 Nigeria DHS is the most recent version of the Nigerian DHS available at the time that we began this analysis, and contained the variable for gestational age as duration of pregnancy. We explained this on Page 5.

3. Please clarify some missing elements in how the multi-level analysis with DHS was conducted. For example, although authors reported that they adjusted the clustering effects in MLM, intra-class correlation coefficients and goodness of fit statistics for model improvement was not reported.

**Response:** In response to this comment, we have added additional information to the text on Page 9: “Births from the same cluster were correlated with an intraclass correlation coefficient (ICC) of 0.05 (likelihood ratio test=31.9). As expected, different births from the same woman were highly correlated with an ICC of 0.42 (likelihood ratio test=249.1) as determined by the three-level linear mixed effect model.”

4. Please explain if a further analysis to examine the association between biomass fuel and a binary outcome of birthweight (underweight vs. normal) is possible to see if the results are similar. If the results are not similar, it should be reported in the manuscript and would enrich the discussion

**Response:** We compared distribution of birth weight as a binary outcome (low birth weight vs. normal) in CEGROMS (Kaduna study) and 2018 Nigerian DHS (replication study) datasets (Table 1).

Table 1. Distribution of low birth weight among term births in CEGROMS and 2018 Nigerian DHS

| **Low birth weight (<2500g)** | **LPG** | **Kerosene** | **Biomass fuel** | **p-value** |
| --- | --- | --- | --- | --- |
| CEGROMS (n=1502) | 57 (4.8) | 2 (1.6) | 13 (7.0) | 0.09 |
| 2018 Nigerian DHS (n=6975) | 87 (5.4) | 70 (5.0) | 285 (7.2) | <0.01 |

We observed that the proportion of infants with low birth weight (LBW) is slightly greater in mothers using biomass fuel compared to LPG or kerosene users in both the primary and replication analysis. However, we could not perform a more robust regression analysis to examine the association between biomass fuel and a binary outcome of birthweight because there are too few cases of LBW among kerosene and biomass fuel users in the CEGROMS dataset. In the comparable analysis in Jiang et al (Ref 16), which had a larger sample size and analyzed birth weight as a binary outcome, they had too few LBW to find a significant association as they did in the preterm using their binary outcome of LBW vs normal weight. When they did their analysis stratified by term or preterm, among the term births they had only seven (7) biomass fuel exposed that were in their case group of low birth weight. In response to this comment, we now point this out in the discussion:

However, power was limited for analysis of biomass among term births with only 7 exposed low birth weight babies.^16^

5. The discussion should be enriched by explaining more in dept some existing interventions, policies and communication strategies to reduce exposure to air pollution, it would be important and interesting for those working in intervention development. Also, a more solid discussion in the research of this area very relevant for the developing countries. Finally, spelling out a little more of these potential implications for practice, will be excellent for awareness on this topic.

**Response:** We have enriched the discussion by explaining in more depth some existing interventions, policies and communication strategies to reduce exposure to indoor air pollution. We have added the following to the discussion

“We recommend that pregnant women attending antenatal care be asked about cooking fuels and provided adequate resources to minimize prenatal biomass exposures.”

And a few sentences later:

Actionable strategies for reducing the presence of indoor pollutants and personal exposures could be developed by combining scientific knowledge about the effectiveness of existing interventions and a social-ecological systems framework: synthesis of existing interventions and literature to elucidate relationships among spatially and otherwise diverse indoor air quality factors.^47^
